# Pelvic Radiotherapy in Rectal Cancer Patients With Synchronous Potentially Treatable Liver Metastases

**DOI:** 10.1002/cnr2.70122

**Published:** 2025-01-23

**Authors:** Yayu Huang, Genwen Chen, Xian Zhang, Yang Qian, Jian Wang

**Affiliations:** ^1^ Department of Radiation Oncology Zhongshan Hospital (Xiamen), Fudan University Xiamen China; ^2^ Xiamen Clinical Research Center for Cancer Therapy Xiamen China; ^3^ Clinical Research Center for Precision Medicine of Abdominal Tumor of Fujian Province Xiamen China; ^4^ Department of Radiation Oncology Zhongshan Hospital, Fudan University Shanghai China; ^5^ Cancer Center, Zhongshan Hospital, Fudan University Shanghai China

**Keywords:** liver metastases, local control, no evidence of disease (NED), radiotherapy, rectal cancer

## Abstract

**Background:**

The optimal management strategy for Stage IV rectal cancer with potentially treatable liver metastases remains controversial, particularly regarding the role of pelvic radiotherapy (RT).

**Aims:**

We intend to investigate the impact of pelvic RT on oncological outcomes of rectal cancer with potentially treatable liver metastasis.

**Methods and Results:**

This retrospective study included 83 patients diagnosed with rectal cancer and synchronous liver metastases from June 2012 to January 2022. All patients underwent radical surgery for rectal cancer and treatment of synchronous liver metastases, as determined by a multidisciplinary team (MDT). We divided the 83 patients into two treatment groups: chemoradiotherapy and surgery (CRT + S) and chemotherapy and surgery (C + S). The CRT + S group (*n* = 40) received pelvic RT, systemic therapy, and liver metastasis treatment. The C + S group (*n* = 43) received systemic therapy and liver metastasis treatment only. A total of 83 patients were analyzed with a median follow‐up of 45 months (range 12–127 months). In the CRT + S group, 48.2% (40/83) of patients underwent chemoradiotherapy, while the C + S group comprised 51.8% (43/83) of patients who received chemotherapy only. The CRT + S group demonstrated significantly longer local recurrence‐free survival compared to the C + S group (median 37.5 vs. 34 months; *p* = 0.011). In addition, patients in the CRT + S group had a longer median overall survival (OS) compared to the C + S group (46.50 vs. 44.0 months; *p* = 0.0497). Notably, achieving no evidence of disease (NED) status after definitive treatment for both primary and liver metastases was associated with improved OS (*p* = 0.008).

**Conclusion:**

This study suggests that the addition of pelvic RT to multimodality therapy for patients with rectal cancer and potentially treatable liver metastases may improve local control and long‐term survival. The findings support the consideration of RT in the clinical management of this patient population.

## 1 Introduction

Colorectal cancer is the third most common cancer and the fourth cause of cancer‐related mortality worldwide [1], with rectal cancer accounting for approximately 40% of cases. Roughly half of patients with colorectal cancer will develop liver metastases (LMs) and 20% of these patients have LMs at first diagnosis [2]. Over the past decade, there have been significant advancements in the management of colorectal liver metastases (CRLM) due to notable improvements in systemic therapy. However, complete resection of both primary tumor and secondary LM can improve survival compared to systemic therapy [3, 4, 5].

The standard treatment for locally advanced rectal cancer (LARC) typically involves neoadjuvant chemoradiotherapy (neo‐CRT). However, local recurrence remains a significant challenge, with a 10‐year cumulative rate of 44.1% reported in a study of 1222 patients who underwent rectal cancer resection. This included 25.6% experiencing local recurrence and 29.9% developing distant metastases [6]. The role of neoadjuvant radiotherapy (RT) in patients with synchronous LMs remains uncertain despite its significant reduction in the risk of local recurrence (hazard ratio [HR]: 0.43, 95% confidence interval [CI]: 0.28–0.66) for LARC. The landmark Dutch trial investigated 1748 patients, with 7% presenting in Stage IV with distant involvement. Patients who only received surgery after a macroscopically complete local resection experienced an 8.2% local recurrence rate. Notably, this was significantly higher compared to the 2.4% local recurrence observed in the group receiving preoperative RT before surgery (*p* < 0.001) [7]. In addition, a retrospective study compared the outcomes of 27 patients with rectal cancer and synchronous LMs who received adjuvant pelvic RT to those of 62 patients who did not receive such treatment. The study revealed a significant reduction in pelvic failure rates among patients with LMs who received adjuvant pelvic RT, with rates of 49.1% and 70.4% observed in the groups receiving and not receiving RT, respectively (*p* = 0.116) [8]. However, no significant difference in overall survival (OS) was observed at 2 years. Previous studies have shown that short‐course radiotherapy (SCRT) combined with upfront chemotherapy followed by delayed surgery is effective and safe for patients with synchronous LMs. In one such study, 41 out of 43 patients (95.3%) achieved synchronous R0 resection, with 3‐year OS and progression‐free survival (PFS) rates of 65.3% and 26.9%, respectively [9]. However, data analysis from the Surveillance, Epidemiology, and End Results (SEER) database revealed that among 6873 patients with stage IV rectal cancer, combined surgery and RT improved 2‐ and 5‐year survival rates compared to RT alone [10]. Notably, achieving a no evidence of disease (NED) status, a status associated with a potential for cure, was associated with further prolonged survival. Therefore, the role of pelvic RT in LARC with LM on survival needs to be further elucidated.

Aggressive multimodal treatment, including chemotherapy, targeted therapy, and surgical resection or radiofrequency ablation (RFA) of detectable lesions, has been shown to result in NED for patients with metastatic colorectal cancer [11]. A previous study demonstrated that achieving NED status was associated with significantly better survival compared to the non‐NED group in patients with advanced rectal cancer experiencing local recurrence and distant metastasis after total mesorectal excision (TME) [12]. These findings suggested that aggressively pursuing NED status through various treatment modalities may improve OS in advanced rectal cancer patients after TME. However, there is still a lack of evidence to prove the role of RT in patients with initially resectable rectal cancer with synchronous LM who are expected to achieve NED status. In this study, we intend to investigate whether pelvic radiation therapy provides survival benefits for rectal cancer patients with synchronous LM. Ultimately, it will provide new evidence for further prospective clinical research and clinical practice.

## 2 Materials and Methods

### 2.1 Patient Eligibility

Between June 2012 and January 2022, 83 patients with rectal cancer and synchronous LM in Shanghai Zhongshan Hospital were enrolled retrospectively in this study. Rectal cancer was defined as a tumor located within 12 cm from the anal verge [7]. Primary and LM pathologies were confirmed as adenocarcinoma; other types of pathologies were excluded. LM was evaluated by abdominal computerized tomography (CT), magnetic resonance imaging (MRI), or positron emission tomography/computed tomography (PET/CT). Synchronous LM was defined as LMs detected at or before the primary tumor. NED was defined as the absence of detectable signs of cancer based on the previously mentioned imaging examinations. This included the successful completion of radical treatment for both the primary tumor and synchronous LM, as determined by a multidisciplinary team (MDT). The definitive treatment for LM could involve surgical resection, RFA, transhepatic arterial chemotherapy and embolization (TACE), or a combination of these. Patients with other metastases were excluded from the study. A total of 83 patients who underwent primary tumor resection and definitive treatment for LM were analyzed. Each patient was evaluated by history, physical examination, blood tests, radiography, and other relevant examinations. The median follow‐up from diagnosis was 45 months (range 12–127 months). This study was approved by the Ethics Committee of Shanghai Zhongshan Hospital.

### 2.2 Treatment Profiles

Decisions regarding resection of primary and metastatic lesions were made on an individual patient basis based on an MDT that included colorectal and liver surgeons, radiation oncologists, medical oncologists, radiologists, and pathologists, according to the patient's wishes. All patients received TME for the primary tumor, while the LMs received surgical tumor resection, RFA, TACE, or a combination. Hepatic resection was performed in patients whose tumor was deemed resectable by the MDT based on the tumor's location, size, number, liver function, and Eastern Cooperative Oncology Group (ECOG) performance status. RFA was regarded as an alternative treatment for tumors less than 3 cm in size and tumors located near large blood vessels or bile ducts. TACE was used as an adjunctive therapy for LMs.

All patients received standard systemic chemotherapy with or without targeted therapy. Chemotherapy regimens included 5‐fluorouracil (5‐FU) + leucovorin (LV) + oxaliplatin (FOLFOX), capecitabine + oxaliplatin (CAPEOX), 5‐FU + LV + irinotecan (FOLFIRI), irinotecan + oxaliplatin + 5‐FU/LV (FOLFOXIRI), capecitabine + irinotecan (CapIRI), irinotecan + vermofenil + cetuximab (VIC), and raltitrexed + oxaliplatin. Targeted therapy included cetuximab and bevacizumab. These were used with one of the above regimens, which were determined based on the gene status of *KRAS*, *NRAS*, and *BRAF*.

The RT strategy included both neoadjuvant and adjuvant chemoradiotherapy regimens. Neoadjuvant CRT consisted of a total pelvic dose of 45–50 Gy (with an optional boost of 5.4 Gy delivered in three fractions) administered using conventional fractionation (1.8–2 Gy per fraction) over 25–28 fractions (long‐course CRT). The boost volume encompassed the tumor, mesorectum, anastomosis, and presacral area. The timing of RT was mainly determined according to the results of the MDT discussion and the patient's wishes.

**FIGURE 1:**
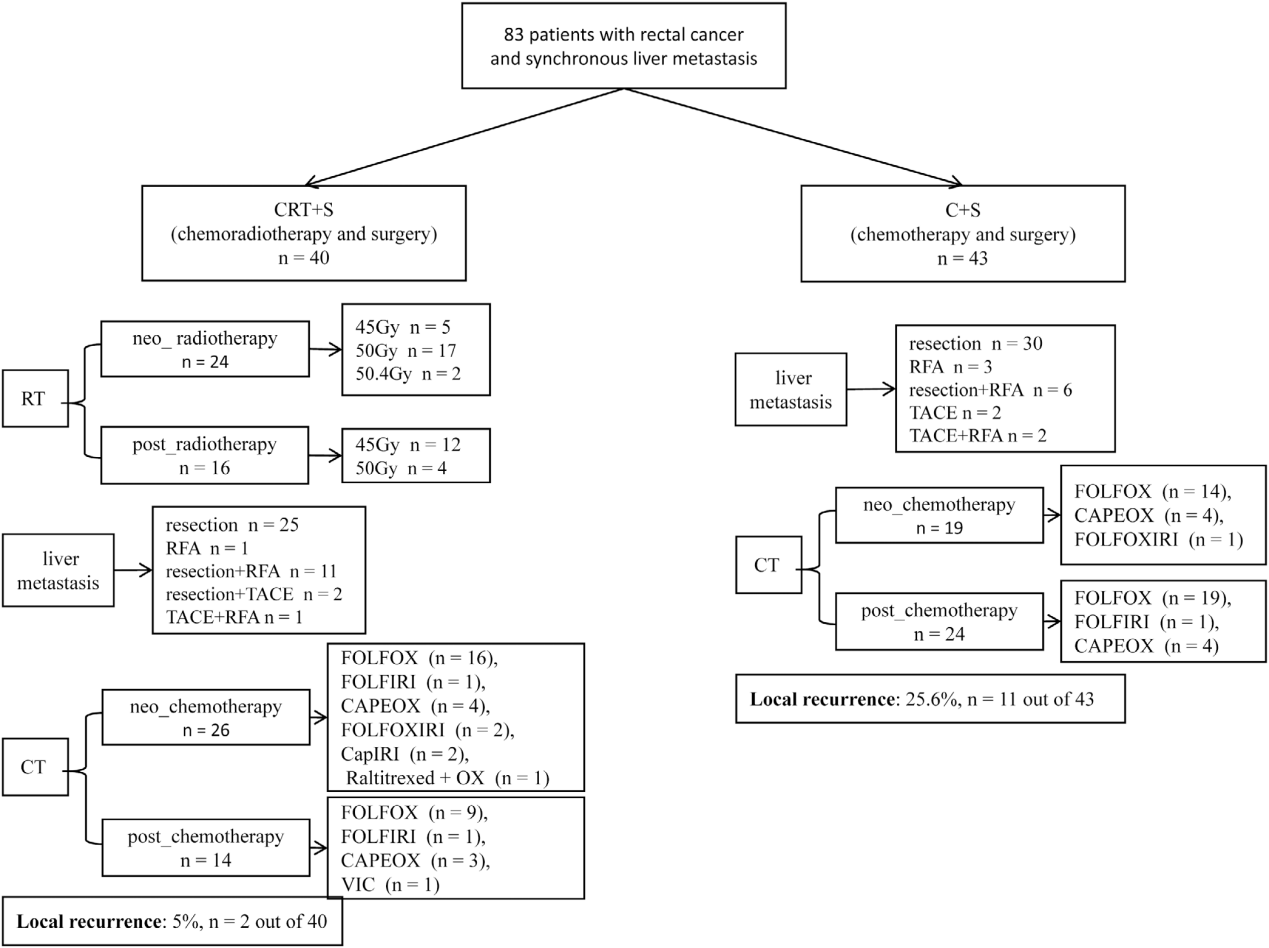
The grouping situation and treatment information of patients. CAPEOX, capecitabine + oxaliplatin; CapIRI, capecitabine + irinotecan; CT, chemotherapy; FOLFIRI, 5‐FU + leucovorin + irinotecan; FOLFOX, 5‐fluorouracil (5‐FU) + leucovorin (LV) + oxaliplatin; FOLFOXIRI, irinotecan + oxaliplatin + 5‐FU/LV; RFA, radiofrequency ablation; RT, radiotherapy; TACE, transhepatic arterial chemotherapy and embolization; VIC, irinotecan + vermofenil + cetuximab.

The grouping and treatment information of patients is shown in Figure 1.

### 2.3 Statistical Analysis

The Kaplan–Meier method was used for the survival analysis. The log rank test was utilized to assess the significance of the difference in survival between the groups. OS was defined as the time from diagnosis to death due to cancer. Patients who were still alive at the end of the follow‐up period were censored. PFS was defined as the time elapsed from diagnosis to either disease progression or death. Pelvic failure‐free survival (PFFS) was defined specifically as the time interval following primary tumor resection until relapse within the pelvic cavity.

The *χ* ^2^ test distinguished differences in these variables between patients who received RT and those who did not. Furthermore, the prognostic impact of RT and clinicopathological factors on patient survival was investigated using the Cox proportional hazards model, conducted in both univariate and multivariate settings. Variables with a *p* value less than 0.05 in the univariate analysis were incorporated into the multivariable competing‐risks regression model for further assessment. HRs were reported with 95% CIs. A *p* < 0.05 was considered as statistically significant. Statistical analyses were performed using the R (R‐studio) and Statistical Package for Social Sciences (SPSS 22.0; SPSS Inc., Chicago, IL).

## 3 Results

### 3.1 Patient and Tumor Characteristics

This study retrospectively investigated 83 patients with rectal cancer and synchronous LMs. All received systemic chemotherapy with or without targeted therapy (cetuximab or bevacizumab) and therapy for LMs. The majority were male (*n* = 51, 61%) with a median age of 56 years at diagnosis (range 22–81). There were no significant differences in baseline characteristics between the RT and no‐RT groups. Table 1 provides a detailed overview of the patients' baseline characteristics.

**TABLE 1:** Demographic and clinical characteristics of 83 patients with rectal cancer and synchronous resectable liver metastases.

Variables	CRT + S (*n* = 40)	C + S (*n* = 43)	*p*
Age, median (range)	56 (22–74)	54 (30–81)	0.733
Gender			
Female	13 (32.5)	19 (44.2)	0.274
Male	27 (67.5)	24 (55.8)	
Distance to the anal verge			
≤ 5 cm	14 (35.0)	16 (37.2)	0.834
> 5 cm	26 (65.0)	27 (62.8)	
Initial T stage			
cT1‐3	28 (70.0)	33 (76.7)	0.487
cT4	12 (30.0)	10 (23.3)	
Initial N stage			
Positive	27 (67.5)	21 (48.8)	0.085
Negative	13 (32.5)	22 (51.2)	
MRF			
Positive	20 (50.0)	20 (46.5)	0.751
Negative	20 (50.0)	23 (53.5)	
Liver metastases, median (range)	4 (1–19)	2 (1–18)	0.762
*RAS*/*RAF* status			
Wild‐type	17 (43%)	17 (40%)	0.784
Mutation	23 (58%)	26 (60%)	
MMR			
pMMR	38 (95%)	41 (95%)	1.000
dMMR	2 (5.0%)	2 (4.7%)	
Targeted therapy			
Yes	28 (70.0)	27 (62.8)	0.488
No	12 (30.0)	16 (37.2)	
NED status after treat			
Yes	26 (65.0)	27 (62.8)	0.508
No	14 (35.0)	16 (37.2)	

Abbreviations: LN, lymph node; MMR, mismatch repair; MRF, mesorectal fascia; NED, no evidence of disease.

Upon initial diagnosis, 36% (*n* = 30) of the patients presented with low rectal cancer (anal verge‐to‐tumor distance less than 5 cm). Twenty‐seven percent (*n* = 22) of the patients had advanced tumors, categorized as clinical T4. While 42% (*n* = 35) were free of regional lymph node metastasis, 58% (*n* = 48) had varying degrees of lymph node involvement (the initial T stage and N stage were based on clinical, imaging and noninvasive or minimally invasive diagnostic procedures). Nearly half (48%, *n* = 40) exhibited involvement of the mesorectal fascia (MRF) by the tumor. The median number of LMs was 4 (range 1–19). Importantly, 59% of the patients were *RAS* or *BRAF* mutations, and 4.8% (*n* = 4) of patients were mismatch repair‐deficient (dMMR). Overall, 54% (*n* = 45) of the patients received neoadjuvant chemotherapy (neo‐CT), and 66% (*n* = 55) of patients received targeted therapy, including anti‐EGFR or anti‐VEGFR agents. No significant differences were observed in the baseline characteristics of the two groups, which are detailed in Table 1.

We divided the 83 patients into two treatment groups: chemoradiotherapy and surgery (CRT + S) and chemotherapy and surgery (C + S). The CRT + S group (*n* = 40) received pelvic RT, systemic therapy, and treatment for LMs. The C + S group (*n* = 43) received systemic therapy and treatment for LMs only. In CRT + S group, 40 patients received pelvic RT, 24 of whom received neoadjuvant RT and 16 of whom received adjuvant RT. The radiation dose was 45–50.4 Gy/25–28 fractions. In total, 40 patients received chemotherapy, 26 of whom received neo‐CT and 14 of whom received postoperative chemotherapy. In the C + S group, 43 patients received chemotherapy, 19 of whom received neo‐CT and 24 of whom received postoperative chemotherapy. More detailed information is described in Figure 1.

### 3.2 Efficacy and Survival

All patients underwent TME and treatment of LMs. For the entire population, the median OS was 45 months (range 12–127 months). Forty‐one patients died, and 42 patients remained alive at the last follow‐up. Among the 43 patients who did not receive RT, 60.5% (*n* = 26) died during the follow‐up period, while 39.5% (*n* = 17) were alive without having received RT.

**FIGURE 2:**
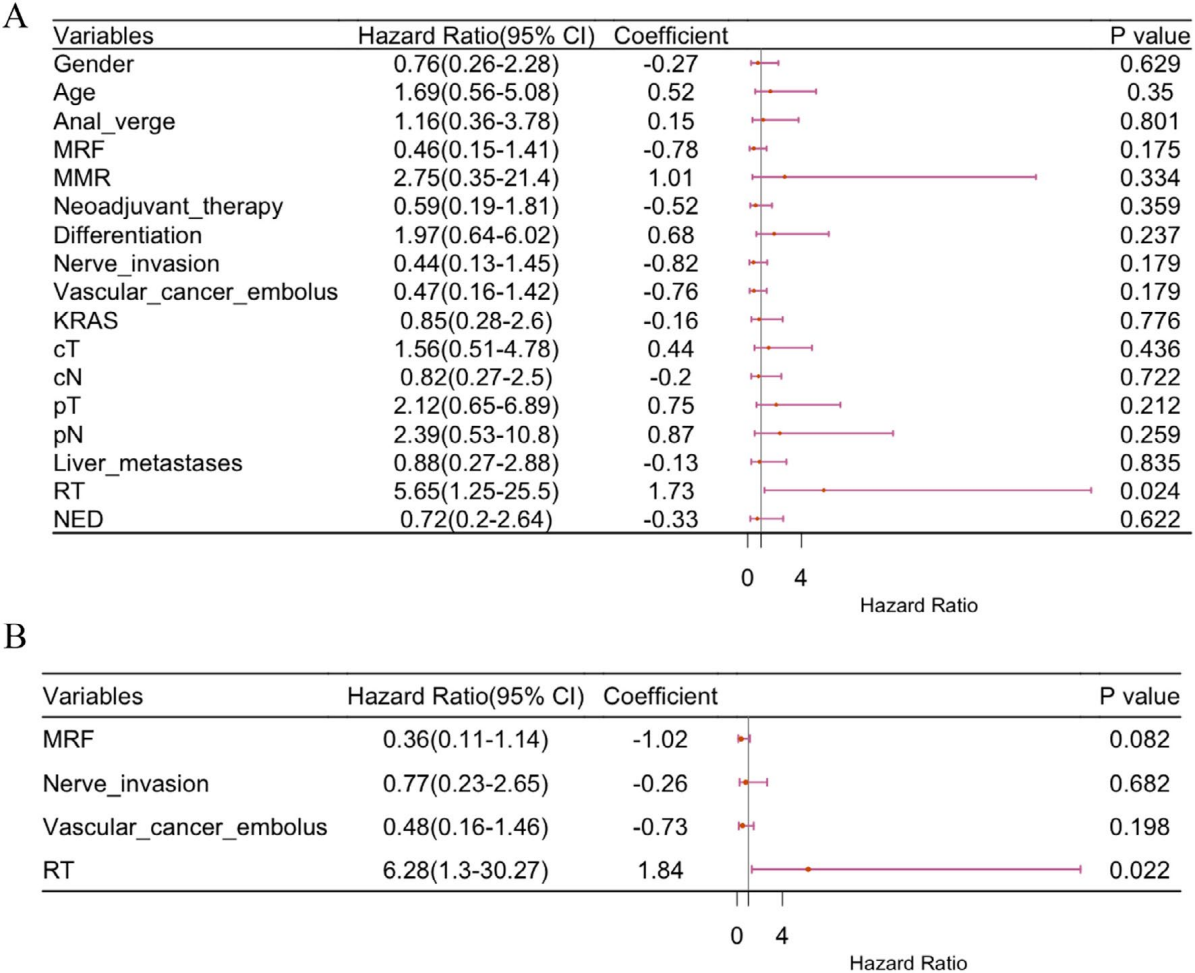
The forest plot for univariate (A) and multivariate (B) Cox regression analysis in local recurrence‐free survival. The figure displays the HR and their 95% confidence intervals for each covariate included in the Cox proportional hazards model.

RT has been reported to reduce pelvic recurrence. We performed univariate analysis, which revealed that RT was associated with a lower rate of pelvic recurrence (*p* = 0.024, Figure 2A). MRF status, nerve invasion, vascular cancer embolus, and RT (all with *p* < 0.2) were included in the multivariate analysis. Only RT remained significantly associated with a lower rate of pelvic recurrence (Figure 2B). Local recurrence was more frequent in the C + S group (25.6%, *n* = 11 of 43) compared to the CRT + S group (5%, *n* = 2 of 40) (Figure 1). A significant difference in local recurrence rates was observed between the groups. Patients who underwent RT demonstrated significantly prolonged local recurrence‐free survival in comparison to those who did not receive this treatment. The median duration of local recurrence‐free survival was 34 months for the group receiving C + S and 37.5 months for the group receiving CRT + S (*p* = 0.011; Figure 4A).

**FIGURE 3:**
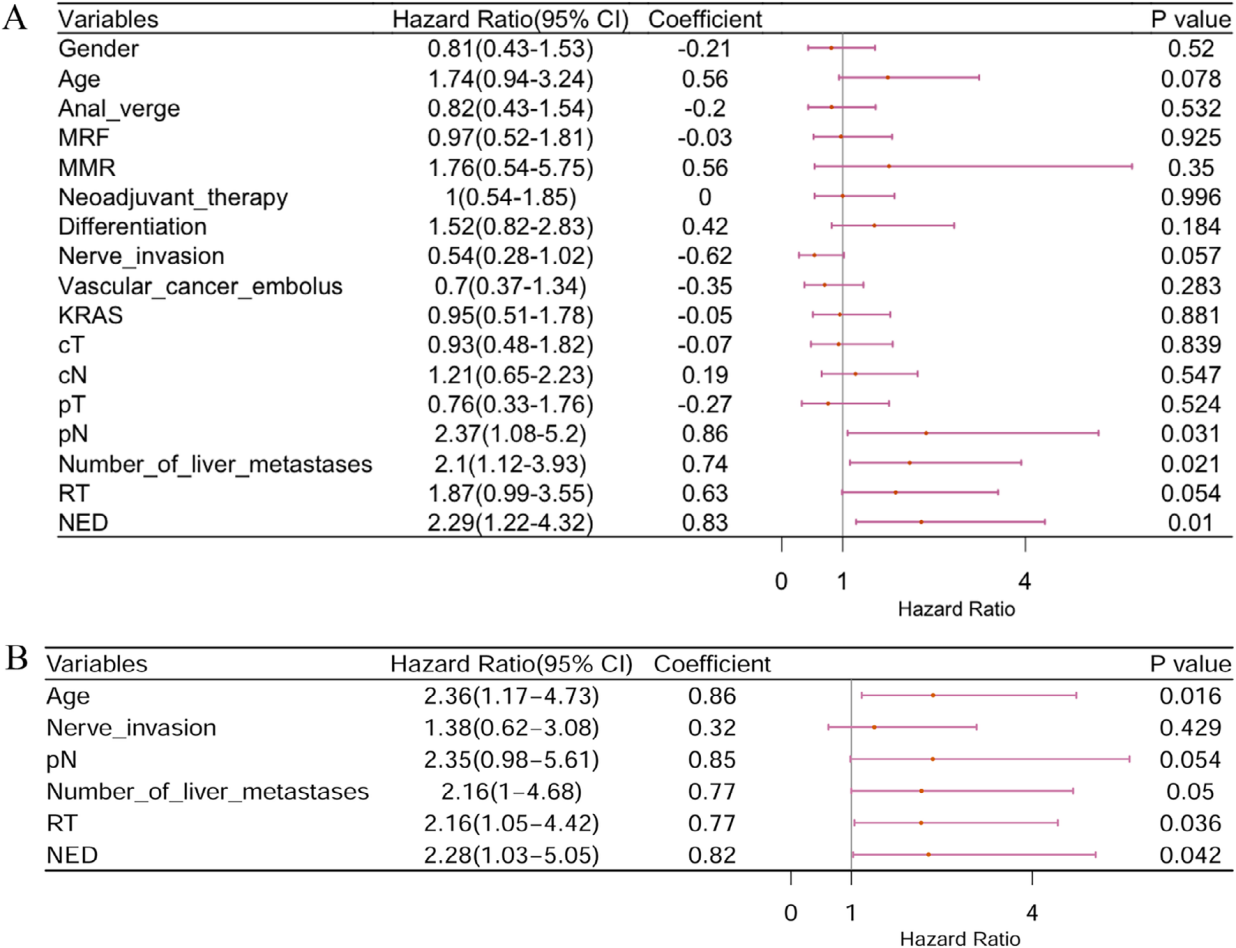
The forest plot for univariate (A) and multivariate (B) Cox regression analysis in overall survival. The figure displays the HR and their 95% confidence intervals for each covariate included in the Cox proportional hazards model.

**FIGURE 4:**
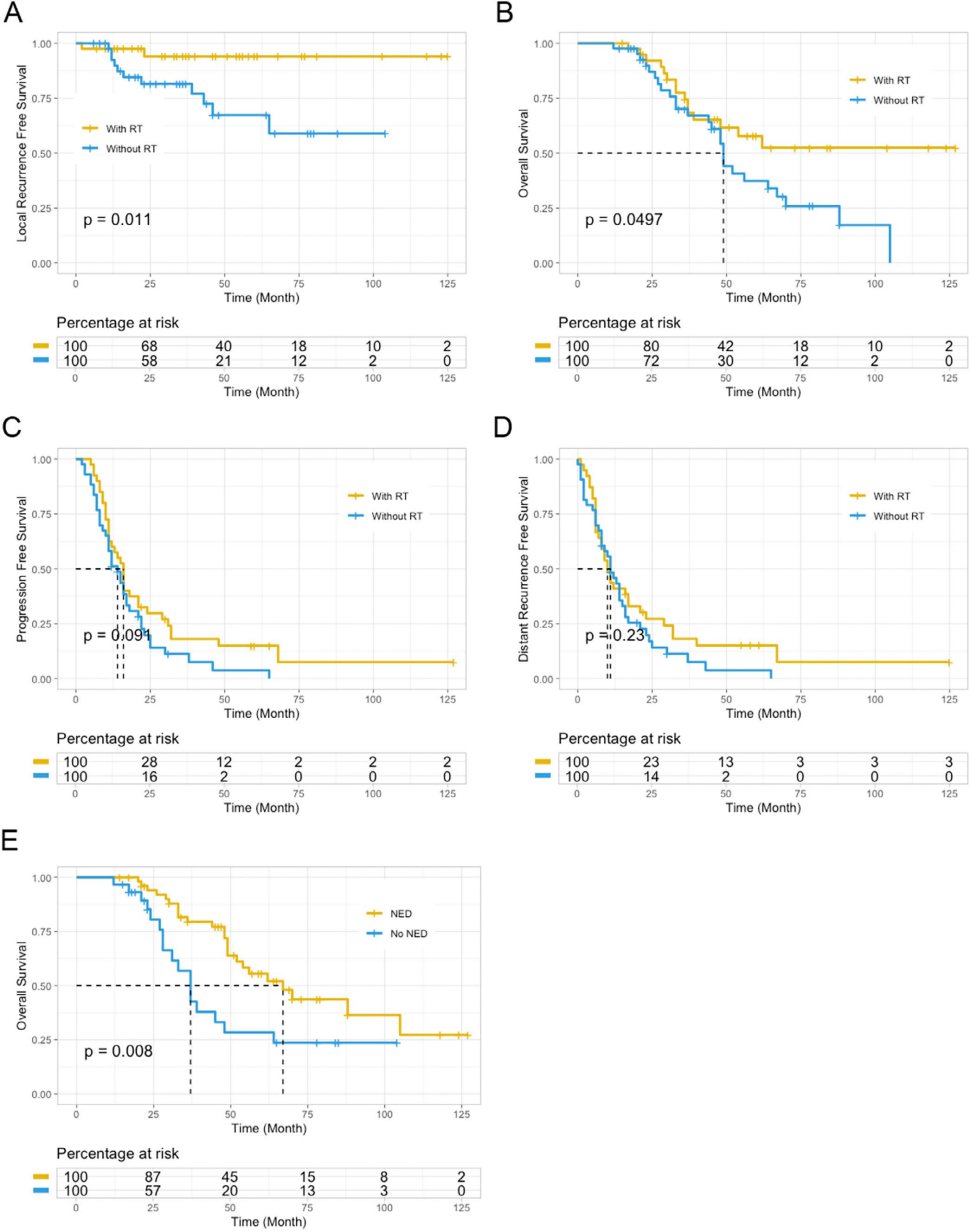
Kaplan–Meier analysis of local recurrence‐free survival (A), overall survival (B), progression‐free survival (C), and distant recurrence‐free survival (D) for the patients who received RT, and those who did not. (E) Kaplan–Meier analysis of overall survival for the patients who achieved NED status, and those who did not.

To investigate whether RT also affects OS, we performed Cox regression analysis and survival analysis. Univariate analysis showed that positive pathological lymph node stage (N+), more than four LMs, and not achieving NED status after treatment were associated with poorer OS. Age (above 60 years), nerve invasion, and not receiving RT were also associated with poorer OS, though with borderline *p* values (Figure 3A). Therefore, factors with a *p* < 0.1 were included in the multivariate analysis of OS. Age (above 60 years), more than four LMs, RT, and NED status were identified as independent prognostic factors for OS (Figure 3B). Figure 4B shows the impact of RT on OS. The CRT + S group had a longer median OS (46.5 months) compared to the C + S group (44.0 months) (*p* = 0.0497). However, the addition of RT did not significantly impact PFS or distant progression‐free survival (DPFS). In the C + S group, the median PFS was 12 months compared to 16 months in the CRT + S group. Similarly, the median DPFS was 11 months in the C + S group compared to 10 months in the CRT + S group (Figure 4C,D).

We further analyzed the downstaging of the primary tumor in patients who received preoperative neo‐CRT, as well as the impact of neo‐CRT on the regression of LMs. Among the 24 patients who received neo‐CRT, 12 patients (50%, *n* = 12 of 24) experienced downstaging of the tumor after chemoradiotherapy. In contrast, among the patients who only received neo‐CT, four patients (21.1%, *n* = 4 of 19) experienced downstaging of the tumor after treatment (*p* = 0.05). The difference was statistically significant, but the *p* value was borderline. Notably, among the patients who only received neo‐CT, there was one patient with a positive surgical margin. Among the 24 patients who received neo‐CRT, 19 patients (79.2%, *n* = 19 of 24) achieved a reduction in LMs before local treatment of the liver lesions. In contrast, among the 19 patients who only received neo‐CT, 15 patients (78.9%, *n* = 15 of 19) also achieved a reduction in LMs. The results indicate that the addition of RT did not provide distant control of LMs (*p* = 0.995).

In addition, we explored the proportion of patients who achieved NED status after receiving definitive treatment for both the primary tumor and LMs. Following LM treatment, the NED rate was 65.0% (*n* = 26) in the CRT + S group and 62.8% (*n* = 27) in the C + S group, respectively (*p* = 0.508; Table 1). Patients who achieved NED status had a longer OS compared to those who did not (*p* = 0.008; Figure 4F). This suggests that achieving NED status is associated with prolonged survival for patients with treatable LMs.

## 4 Discussion

Compared to other cancers, colorectal cancer displays a higher propensity for LM due to the drainage of blood through the portal venous system [13]. Studies have shown that achieving NED status through intensive treatment in these patients is associated with significantly improved survival outcomes [10]. Therefore, we investigated the combination of pelvic RT with LM treatment for patients with potentially treatable rectal cancer, aiming to achieve a NED status. However, while our study suggested that pelvic RT was beneficial for the downgrade of pelvic tumor T stage, it did not demonstrate a clear impact on patients achieving NED status or on the effective control of LMs. For patients with LMs, achieving a state of NED primarily depends on the control of LMs. Pelvic RT, as a local treatment method, mainly serves to reduce tumor burden and minimize local recurrence in the pelvic area. Some studies suggested that RT can stimulate the body's immune system, enhancing immune recognition and attack on tumor cells, thereby affecting tumors in other parts of the body. However, more clinical studies are needed to confirm.

The National Comprehensive Cancer Network guidelines advocate for systemic chemotherapy and target therapy as the mainstay treatment for patients with rectal cancer and unresectable LMs. Pelvic RT is only recommended if the metastases become resectable. Similarly, perioperative chemotherapy and pelvic RT are suggested for patients with resectable LMs, but high‐level evidence specifically supporting the benefit of pelvic RT in this context is still lacking. Multiple retrospective studies have investigated the use of SCRT (5 Gy × 5 fractions) with delayed surgery, if feasible, in patients with unresectable rectal cancer with or without synchronous distant metastases. These studies suggest that the 5 Gy × 5 fractions schedule is well‐tolerate [14, 15, 16]. A previous study reported that adjuvant pelvic RT in a cohort of 27 patients with LARC and synchronous distant metastases reduced 2‐year PFFS rates but did not improve 2‐year OS [8]. Notably, a prospective study involving 32 patients with LARC and synchronous LMs evaluated the efficacy and safety of SCRT combined with mFOLFOX6 chemotherapy and surgery. The regimen was found to be safe, with 54% of patients (17/32) achieving primary tumor downstaging and 63% (20/32) achieving R0 resection [17]. In our study, patients who received pelvic RT demonstrated improved pelvic recurrence control compared to those who did not. These findings support the potential benefit of pelvic RT for rectal cancer with synchronous distant metastases and warrant further investigation through prospective studies.

Liver resection offers hope for long‐term survival in patients with LMs. Studies report that approximately 30% of these patients are alive at 5 years after surgery, with two‐thirds achieving NED status. However, the prognosis for patients who do not undergo radical surgery remains poor [2]. Encouragingly, aggressive treatment approaches have shown promising results for advanced rectal cancer with liver involvement. In patients with locally advanced disease and unresectable metastases, chemoradiotherapy followed by surgery to remove all lesions has been linked to improved survival [18]. Similarly, for patients with potentially resectable metastases, completing neoadjuvant chemoradiation, resection/ablation, and adjuvant chemotherapy significantly improves 3‐year OS and PFS compared to those who do not complete treatment, with manageable side effects [19]. The study reported a 74.4% versus 39.2% 3‐year OS for the treatment group compared to the control group, and 45.5% versus 30.5% 3‐year PFS in the same groups. Furthermore, patients with synchronous LMs who can undergo SCRT, systemic therapy, and local treatment of both primary and metastatic lesions also demonstrate favorable survival rates. The median OS was 51.5 months for those completing the treatment compared to 15.1 months for those who did not [20]. Another retrospective study investigated the efficacy of neo‐CT and SCRT with delayed surgery. This approach demonstrated promising results, achieving a high rate of pathologic downstaging of the primary tumor and R0 resection of LMs, leading to improved PFS compared to the palliative treatment group [21]. Our study also suggests that patients who underwent radical treatment for both the primary tumor and LMs and achieved NED status had favorable OS. Notably, among patients who achieved NED, 18.9% (*n* = 10) experienced local recurrence, and 84.9% (*n* = 45) experienced distant metastasis recurrence. These findings demonstrate the potential benefits of comprehensive treatment strategies that combine surgery, RT, and systemic therapy for patients with advanced rectal cancer and LMs.

Several Phase II clinical trials have investigated the potential benefits of pelvic RT for patients with rectal cancer and distant metastases. In one study, 50 patients received SCRT followed by neoadjuvant therapy with bevacizumab and CAPEOX chemotherapy. Subsequently, 36 patients underwent radical surgery. After a median follow‐up of 8.1 years, the median OS was 3.8 years, and 32% of patients remained alive. However, 2 (5.6%) experienced local recurrence and 29 (80.6%) experienced distant recurrence [22, 23]. Another prospective Phase II trial investigated the combination of pelvic RT and first‐line chemotherapy, which is a standard treatment, in patients with rectal cancer and synchronous unresectable distant metastases. The trial reported a median OS of 17.5 months and a median PFS of 12 months for the 32 enrolled patients. Notably, patients who received definitive therapy (including both RT and surgery) had a significantly improved OS compared to those who received only nondefinitive therapy (*p* = 0.045), while there was no significant difference in PFS between the groups (*p* = 0.274) [24]. This finding aligns with our study, where patients who received pelvic RT and radical treatment of all tumor lesions had a favorable OS compared to those who did not receive RT. However, the borderline *p* value for this finding may be due to our limited sample size. We acknowledge that the limited sample size (*n* = 40 in CRT + S and *n* = 43 in C + S) reduces statistical power and increases the potential for Type II errors, which may contribute to the borderline *p* values and the lack of significance in certain outcomes. Larger studies are needed to validate our results and draw more definitive conclusions.

However, there is currently no consensus on the sequence of local treatment for pelvic lesions and the management of LMs. In response to this challenge, European countries have explored the “liver‐first approach” for treating rectal cancer with synchronous LMs. This approach involves administering systemic chemotherapy followed by liver resection and then proceeding with preoperative chemoradiotherapy and primary tumor resection [25, 26].

Our study has several limitations. First, the definition of treatable LMs remains challenging due to its dependence on non‐tumoral liver conditions and anticipated remnant liver volume. Different centers may adopt varying definitions based on their experience, leading to an element of subjectivity in this assessment [27]. Consequently, despite all 83 patients receiving TME and treatment of LMs, 30 of them (34.9%) were unable to achieve NED status. In our study, the MDT discussed the assessment of LM resection [28], aiming to enhance objectivity to the extent possible. Second, as a retrospective study, it is inherently susceptible to selection bias. Therefore, a prospective randomized trial is necessary to confirm our findings.

## Author Contributions

All authors contributed to the conception and design of the study. Material preparation, data collection, and analysis were conducted by Yayu Huang, Genwen Chen, Xian Zhang, and Yang Qian. The first draft of the manuscript was authored by Yayu Huang and Genwen Chen, with all authors providing feedback on earlier versions. Finally, all authors read and approved the final manuscript.

## Conflicts of Interest

The authors declare no conflicts of interest.

## Data Availability

The data that support the findings of this study are available on request from the corresponding author. The data are not publicly available due to privacy or ethical restrictions.
